# Muscle regeneration in gilthead sea bream: Implications of endocrine and local regulatory factors and the crosstalk with bone

**DOI:** 10.3389/fendo.2023.1101356

**Published:** 2023-01-23

**Authors:** Aitor Otero-Tarrazón, Miquel Perelló-Amorós, Violeta Jorge-Pedraza, Fatemeh Moshayedi, Albert Sánchez-Moya, Isabel García-Pérez, Jaume Fernández-Borràs, Daniel García de la serrana, Isabel Navarro, Josefina Blasco, Encarnación Capilla, Joaquin Gutierrez

**Affiliations:** Department of Cell Biology, Physiology and Immunology, Faculty of Biology, University of Barcelona, Barcelona, Spain

**Keywords:** skeletal muscle, injury, regeneration, GH-IGFs axis, proteolytic systems, myogenesis, crosstalk, bone

## Abstract

Fish muscle regeneration is still a poorly known process. In the present study, an injury was done into the left anterior epaxial skeletal muscle of seventy 15 g gilthead sea bream (*Sparus aurata*) juveniles to evaluate at days 0, 1, 2, 4, 8, 16 and 30 post-wound, the expression of several muscle genes. Moreover, transcripts’ expression in the bone (uninjured tissue) was also analyzed. Histology of the muscle showed the presence of dead tissue the first day after injury and how the damaged fibers were removed and replaced by new muscle fibers by day 16 that kept growing up to day 30. Gene expression results showed in muscle an early upregulation of *igf-2* and a downregulation of *ghr-1* and *igf-1*. Proteolytic systems expression increased with *capn2* and *ctsl* peaking at 1 and 2 days post-injury, respectively and *mafbx* at day 8. A pattern of expression that fitted well with active myogenesis progression 16 days after the injury was then observed, with the recovery of *igf-1*, *pax7*, *cmet*, and *cav1* expression; and later on, that of *cav3* as well. Furthermore, the first days post-injury, the cytokines *il-6* and *il-15* were also upregulated confirming the tissue inflammation, while *tnfα* was only upregulated at days 16 and 30 to induce satellite cells recruitment; overall suggesting a possible role for these molecules as myokines. The results of the bone transcripts showed an upregulation first, of *bmp2* and *ctsk* at days 1 and 2, respectively; then, *ogn1* and *ocn* peaked at day 4 in parallel to *mstn2* downregulation, and *runx2* and *ogn2* increased after 8 days of muscle injury, suggesting a possible tissue crosstalk during the regenerative process. Overall, the present model allows studying the sequential involvement of different regulatory molecules during muscle regeneration, as well as the potential relationship between muscle and other tissues such as bone to control musculoskeletal development and growth, pointing out an interesting new line of research in this group of vertebrates.

## 1 Introduction

The gilthead sea bream (*Sparus aurata*, Linnaeus 1758) is one of the most important species in the Mediterranean aquaculture of the last decades as it represents a source of energy and proteins of high quality and thus, it has a high commercial value (1). However, there are still areas of their physiology that are not well described; therefore, more research is needed to optimize their growth and development in terms of farming.

In contrast to other vertebrate groups, some fish species exhibit indeterminate growth, which means that they can grow in length and weight beyond the maturation stage for as long as they live (2). Most of this growth is due to an increase in somatic tissue, specifically skeletal muscle, and implies new muscle cells recruitment to increase the number of muscle fibers (hyperplasia), as well as the increase in size of pre-existing fibers (hypertrophy). These phenomena are known to be an important determinant of the characteristics of flesh texture and, from an aquaculture perspective, muscle quality is of main interest (3, 4).

The growth hormone (Gh) and insulin-like growth factors (Igfs) axis is the most important endocrine system controlling many physiological processes of the body (5). The Gh is the central regulatory molecule of somatic growth in vertebrates, including teleost fish, and although its main target is the liver it can exert significant direct effects on other tissues such as skeletal muscle. However, it is well known that most of the effects of Gh on growth are indirect, through the Igfs (6). The large tissue distribution of Igfs-producing cells including their receptors, together with the extensive distribution of Gh receptors (Ghrs), make this mediation possible. Many studies, including several in fish, have demonstrated systemic effects as well as the existence of local paracrine/autocrine actions of Gh and Igfs (7, 8). In muscle, Igfs directly stimulate a variety of signals including proliferation and differentiation of satellite cells (SCs), hypertrophy of myoblasts, and inhibition of cell atrophy. In gilthead sea bream, it has been shown that Igf-2 alone appears to be an important stimulator of proliferation, while Igf-1 seems to have a synergetic activity with Gh, playing different roles in the control of myogenesis (9). Moreover, in the same species, three splice variants have been described (*igf-1a*, *igf-1b* and *igf-1c*) with differential responses depending on the physiological or stimulatory condition (10). Igfs exert these actions through the binding and activation of their cellular receptors and in turn multiple intracellular signal transduction cascades, including the phosphatidylinositol 3-kinase (Pi3k)-Akt-Tor cascade, which controls an important gene expression program (11).

The process of muscle generation, known as myogenesis, consists in that SCs, the muscle stem cells that can be recognized for the expression of Pax3/7 (12), proliferate and differentiate into myoblasts that fuse together (i.e., hyperplasia) or with pre-existing fibers (i.e., hypertrophy) to form multinucleated myofibers. Myogenesis is regulated by the myogenic regulatory factors (Mrfs), a set of four transcription factors that are expressed sequentially being Myf5 and MyoD the first to be activated controlling SCs activation and proliferation, and later Myogenin and Mrf4 that coordinate myoblasts differentiation and fusion (13). In adulthood, those processes can also occur in response to challenging conditions that threaten muscle homeostasis. Thanks to the ability of muscle cells to modify both, the amount and types of proteins being synthesized within them, defined as muscle plasticity, it is possible to cope with any stimulus that disrupts the normal condition of the organism (14). In this framework, muscle regulation implies a protein turnover to ensure the correct function of the myofibers, i.e., a balance between protein synthesis and degradation. The main catabolic systems that control muscle proteolysis have been also described in gilthead sea bream and are the ubiquitin-proteasome complex (UbP); calpains, a family of Ca^2+^-dependent proteases formed by catalytic and regulatory small subunits; cathepsins, some of them related to the autophagy-lysosome system; and caspases, the apoptosis protease system (15, 16).

One of the phenomena that can alter normal muscle functions and body homeostasis are plasma membrane disruptions induced by muscle contraction as well as severe damage caused by traumatic injuries or muscle diseases, leading all these processes to fiber rupture. Nevertheless, skeletal muscle presents an intrinsic mechanism capable of regenerating the entire damaged contractile system. The fundamental role in muscle regeneration is played by the SCs resident in the muscle, which are activated in response to various previous stimuli that may come from the tissue itself or from other systems of the organism (17). When necrosis of the affected myofibers occurs because of the trauma, first an inflammatory response is initiated with the release of different cytokines, as the interleukins, from neutrophils, among other immune cells (18, 19). These induce macrophages infiltration and activate the proteolytic systems to remove all the dead material, followed by an activation of SCs to generate new fibers or to fuse with undamaged fibers. Finally, newly formed fibers maturate and remodel the regenerated muscle to recover its normal functions (20).

In addition, there are other factors that can also have critical roles during myogenesis. Proliferating SCs are characterized by the presence of proliferating cell nuclear antigen (Pcna), which is classically known to stimulate the entry of SCs to the cell cycle (21), and of hepatocyte growth factor receptor (cMet) that turns on also cell proliferation in skeletal muscle (22, 23). On the other hand, two novel muscle-specific fusogens, Myomaker and Myomixer, have been described and could play a fundamental role in the stage in which myoblast fusion takes place. In fact, the lack of some of these two muscle-specific membrane proteins results in an incomplete myogenesis since cells cannot fuse and therefore multinucleated myofibers are not formed (24). Other factors with an important role are caveolae, which are invaginations of the plasma membrane that function as messenger centers in tissues, including skeletal muscle, controlling signal transduction. The transmembrane proteins that enable the formation of these structures are members of the caveolin family (25). Caveolin-1 (Cav-1) was the first member described and was identified as being expressed in quiescent SCs but not in mature myofibers and is downregulated when cells begin to proliferate (26). The other two members of the family are Caveolin-2 (Cav-2) and Caveolin-3 (Cav-3). Characterization of the former revealed that colocalizes with Cav-1 and is co-expressed in the same circumstances. Otherwise, Cav-3, unlike Cav-1, is involved in myoblast differentiation and myotube fusion, so that a deficiency of Cav-3 results in cellular immaturity (27). Finally, another family of proteins essential for the maintenance of skeletal muscle homeostasis in adults are the Wnts, although information about the role of these molecules in fish is very limited. Canonical Wnt signaling regulates the differentiation of myoblasts, while the non-canonical pathway controls the self-renewal of SCs and the growth of muscle fibers (28, 29).

In some teleost fishes, like the gilthead sea bream, and as opposed to mammals, the skeletal system is defined as acellular since the skeleton is generated and maintained by chondrocytes, osteoblasts, and osteoclasts but it lacks osteocytes within the calcified extracellular matrix (ECM) (30, 31). Bone lineage determination and differentiation of osteoblasts from mesenchymal stem cells (MSCs) implies various key regulatory actors including specific transcription factors, mineral availability, and environmental conditions (32). The transcription factor expression pattern that controls osteogenesis was elucidated in gilthead sea bream few years ago and it was demonstrated that there is conservation of transcripts compared to mammals (33). Indeed, Runx2 is crucial for the commitment of the osteoprogenitor cells from MSCs and for the differentiation of osteoblasts; besides it coordinates the expression of other genes, such as *osteopontin* (*op*), *osteonectin* (*on*) and *osteocalcin* (*ocn*), among others, which are key factors regulating the ECM mineralization (34). In addition, it is essential to point out that bone is among the only tissues that has a cell type whose main action is to destroy the own tissue, the osteoclast (35). This function is known as bone remodeling and it is crucial to guarantee mineral homeostasis, having regulatory implications also in other tissues such as the skeletal muscle (36).

Bones represent the attachment location for skeletal muscles and therefore this tissue is also important to cope with threatening conditions. Hence, the musculoskeletal system is a complex interconnected structure (37). Together, the musculoskeletal system enables locomotion and has an essential metabolic role, and the interaction of muscle and bone tissues goes beyond the mechanical and involves a great deal of biochemical communication (38, 39). Both tissues are a relevant source of signaling molecules that can act in an autocrine, paracrine, or endocrine way. It is for these reasons that the functions and processes of each individual tissue are regulated by the other one (40, 41).

In this scenario, the main objective of this work is to perform an *in vivo* study to characterize skeletal muscle regeneration in gilthead sea bream. For this purpose, a mechanical injury will be produced in the muscle and changes in the transcription of key genes involved in the growth regulation of skeletal muscle, including the different systems and molecules mentioned above will be evaluated. In addition, several bone gene markers will be studied to determine the implication of this close-related tissue regulating myogenesis.

## 2 Materials and methods

### 2.1 Experimental design

For the muscle regeneration study, 140 gilthead sea bream (*Sparus aurata*) juveniles (initial body weight = 15.4 ± 3.5 g; initial length = 8.7 ± 0.6 cm (mean ± SEM)) were obtained from a commercial hatchery (Piscimar, Borriana, Spain) and were placed and adapted to the fish facilities of the Faculty of Biology (University of Barcelona). Fish were randomly distributed in three 200 L seawater tanks (45-47 fish/tank). Each tank had a constant flux of 700 L/h in a seawater semi-closed recirculation system with a weekly renewal of 20-30% and a salinity of 35-37 ‰ at a constant temperature of 23 ± 1°C. In the water tank room, there was a photoperiod of 12 h light/dark. Fish were fed *ad libitum* 3 times per day with a commercial diet (Perla, Skretting, Burgos, Spain) and were kept in the described conditions for their acclimatization during 2 weeks before the experiment. Once the study started, all the animals continued being fed with the same protocol as during the adaptation period. The study was carried out following the EU recommendations and the procedures established by the Spanish and Catalan governments. The protocol was approved by the Ethics and Animal Care Committee of the University of Barcelona (CEEA 37/20).

This study aimed to better understand adult muscle regeneration after an injury. To do that, 140 gilthead sea bream were divided into two groups: injured fish (I) and control fish (C) and randomly distributed in three tanks. Specifically, in each seawater tank, 33 fish from the injured group were housed with 14 fish from the control group. First, all the gilthead sea bream juveniles were anesthetized with MS222 (100 mg/L) and then measured and weighed. To identify the fish, a passive integrated transponder (PIT) tag (ID-100A (1.25) Nano transponder; Trovan Electronic Identification Systems, Madrid, Spain) was inserted subcutaneously into the left anterior epaxial muscle just below the first radius. Subsequently, the injury was performed with a 2.108 mm (14G) diameter needle inserted vertically into the left epaxial muscle below the sixth radius to a depth of 1 cm. To know exactly in the future days where the needle was introduced, the tip of the sixth radius was cut, also to the control fish for comparative purposes. Then, the wound was healed with iodine alcoholic solution and the fish were allowed to recover in a separated small tank before being returned to the 200 L tank.

Tissue samples were obtained at days 0, 1, 2, 4, 8, 16 and 30 after the injury. At each time, 16 fish were randomly selected for the study groups. To carry out the samplings, fish were first anesthetized, identified reading the pit tag, weighed to note the changes on body weight and then, blood was drawn to ensure a cruelty-free death. In injured fish (I), a section of the epaxial muscle was removed from the left loin (injured muscle) and the right loin as a self-control for each fish. The tissue size of the muscle extracted was 0.5 cm wide and 1 cm long just below the sixth cut radius. The spinal column was also excised counting as the bone sample of a fish with injury. Moreover, a sample of the vertebral column was also extracted from the control fish (C) to obtain bone control samples. All tissue samples were placed in RNase-free Eppendorf tubes that were stored in liquid nitrogen during sampling and then at -80 °C until the performance of the gene expression analyses.

### 2.2 Histology analysis

Epaxial muscle slices were fitted in histological cassettes and were dehydrated in graded ethanol series and embedded in paraffin. Sections of 7-10 µm were cut with a microtome (pfm, ROTARY 3003, Köln, Germany) and stained with Hematoxylin and Eosin and Sirius Red for collagen staining (42). All preparations were observed under a light microscope and photographed (Olympus PM10SP Automatic Photomicrography System). All reagents for histology staining were purchased from Merck (Merck, Mollet del Vallès, Spain).

### 2.3 Gene expression

#### 2.3.1 RNA extraction and cDNA synthesis

Total RNA was extracted from 100 mg of epaxial muscle or vertebral bone with 1 mL of TRI Reagent Solution^®^ (Applied Biosystems, Alcobendas, Spain) and homogenized on a Precellys^®^ Evolution coupled to a Cryolys cooling unit (Bertin Instruments, Montigny-le-Bretonneux, France). The RNA extraction was performed following the manufacturer’s instructions of the TRI Reagent Solution^®^. The final concentration of each sample was obtained using the Nanodrop 2000 (Thermo Scientific, Alcobendas, Spain) and RNA integrity was confirmed in a 1% agarose gel (*w/v*) stained with SYBR-Safe DNA Gel Stain^®^ (Life Technologies, Alcobendas, Spain).

For RNA-complementary DNA (cDNA) synthesis, 2 μg of total RNA were treated with DNase I Amplification Grade^®^ (Life Technologies, Alcobendas, Spain) to remove all genomic DNA. Reverse transcription was carried out with the First Strand cDNA synthesis Transcriptor Kit ^®^ (Roche, Sant Cugat del Valles, Spain) following the manufacturer’s protocol.

#### 2.3.2 Quantitative real time PCR

The mRNA transcript levels of the genes for both tissues were analyzed by real-time quantitative PCR (RT-qPCR) using the CFX384™ Real-Time System (Bio-Rad, El Prat de Llobregat, Spain). The primers used for each tissue are listed in **Table S1** and the specificity of the amplification, and the absence of non-specific primer bindings were tested *in silico*. First, a dilution curve with a pool of samples was run to confirm primer efficiency and to determine the appropriate cDNA dilution for each assay. All the analyses were performed in triplicate wells using 384-well plates with 2.5 µL of iTaq Universal SYBR Green Supermix (Bio-Rad, El Prat de Llobregat, Spain), 0.25 μL of forward (250 nM) plus reverse (250 nM) primers, 1.25 μL of DEPC water and 1 µL of diluted cDNA for each sample, in a final volume of 5 µL following the conditions described by Salmerón et al. (16). Moreover, three negative controls were also included and ran in duplicate: no template control (NTC), no reverse transcriptase control (RTC), and PCR control (PCR, MilliQ water). The expression level of each gene was calculated with the ΔΔCq method considering the efficiency of each primer pair (43) and was analyzed relative to the geometric mean of the reference genes ribosomal protein s18 (*rps18*), ribosomal protein l27a (*rpl27*) and elongation factor 1 alpha (*ef1a*). The reference genes, the most stable under different conditions, were confirmed with the geNorm algorithm. Both, reference genes stability and relative expression of the target genes were determined using the Bio-Rad CFX Manager Software v. 3.1 (Hercules, CA, USA). Finally, to specifically determine that the changes in relative gene expression were due to the injury, the expression results of the injured samples (i.e., left loin and column of injured fish) were normalized by the gene expression values of the corresponding control samples (i.e., right loin of injured fish and column of control fish).

### 2.4 Statistical analyses

Data were analyzed using IBM^®^ SPSS^®^ Statistics v.25 (IBM, Armonk, NY, USA) and were presented as mean ± standard error of the media (SEM). Normal distribution was analyzed using the Shapiro-Wilk test and homogeneity of the variances (homoscedasticity) was assessed with Levene’s test. Differences were tested by one-way analysis of variance (ANOVA) and the *post-hoc* Tukey HSD. If necessary, the nonparametric Kruskal Wallis test and the *post-hoc* Games-Howell were used. Additionally, one-way ANOVA was performed to verify that the tank did not influence the measured parameters. Statistical differences were considered significant when p < 0.05. Data were plotted using GraphPad Prism^®^ v. 7 (GraphPad Software, La Jolla, CA, USA, www.graphpad.com).

## 3 Results

### 3.1 Histological characterization during muscle regeneration

**Figure 1:**
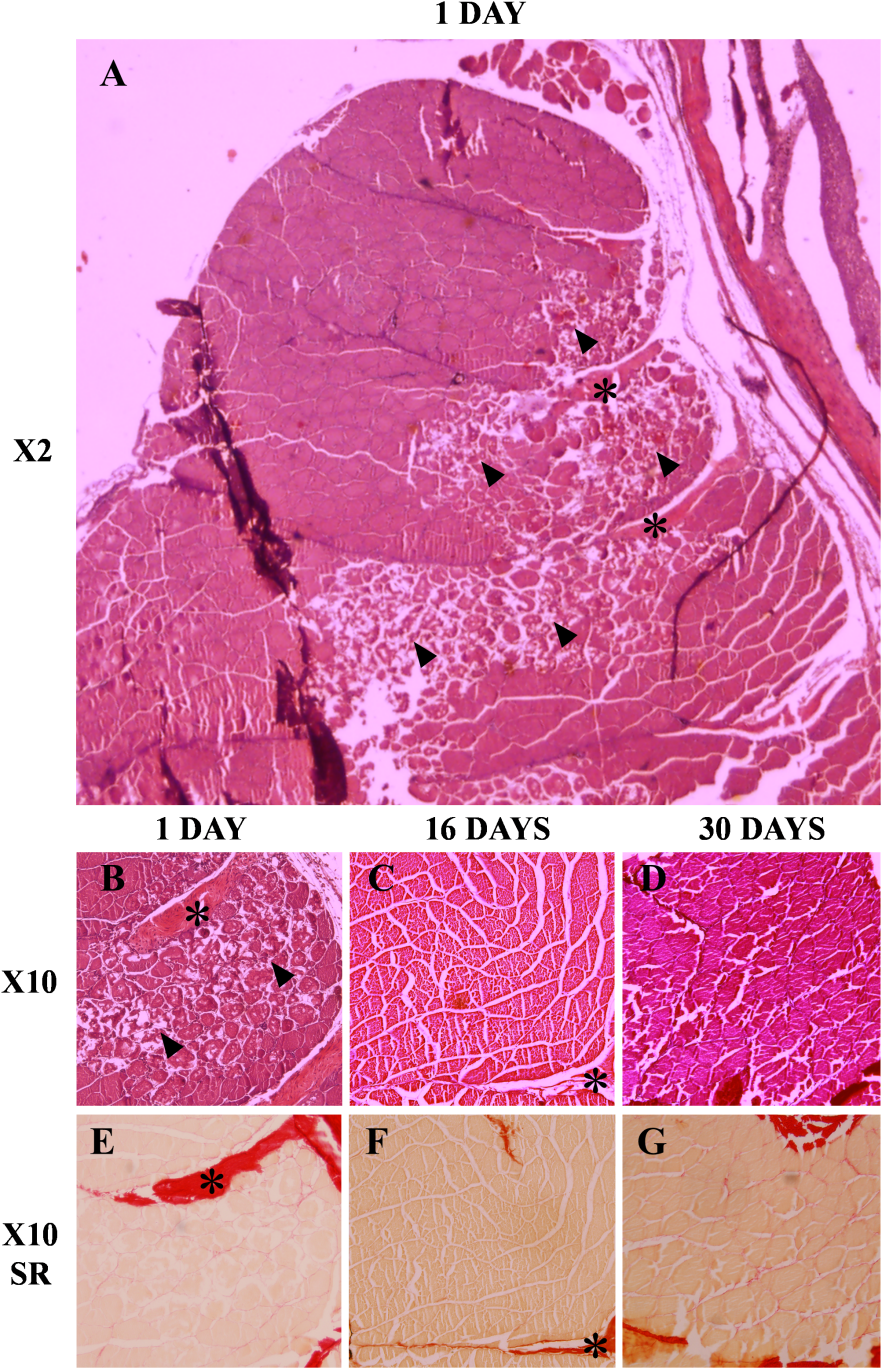
Histological evaluation of the muscle regeneration process at 1, 16 and 30 days post-injury. Muscle sections (7-10 µm) were stained with hematoxylin/eosin **(A-D)** and Sirius red **(E-G)**. The arrowheads mark the presence of muscle fibers in the process of necrosis and the asterisks mark the myoseptum.

The qualitative analysis of the injured muscle tissue samples showed that on day 1 it was possible to observe the presence of dead fibers beginning the process of necrosis in the area where the injury occurred (**Figures 1A, B**). Additionally, an increase in collagen was observed in the myoseptum of the injured area (**Figure 1E**). Later, at day 16, it was no longer possible to find any trace of injured fibers and instead new healthy fibers were observed, in addition to a reduction in the amount of collagen in the myoseptum (**Figures 1C, F**). Finally, in the muscle samples at day 30 neither there were signs of injury nor increased collagen in the myoseptum. Furthermore, it could be seen how the new fibers formed in the injured area were more similar in size to the fibers of unaffected regions showing the entry of the cells into the maturation stage (**Figures 1D, G**).

### 3.2 Expression of genes from the Gh-Igfs axis during muscle regeneration

**Figure 2:**
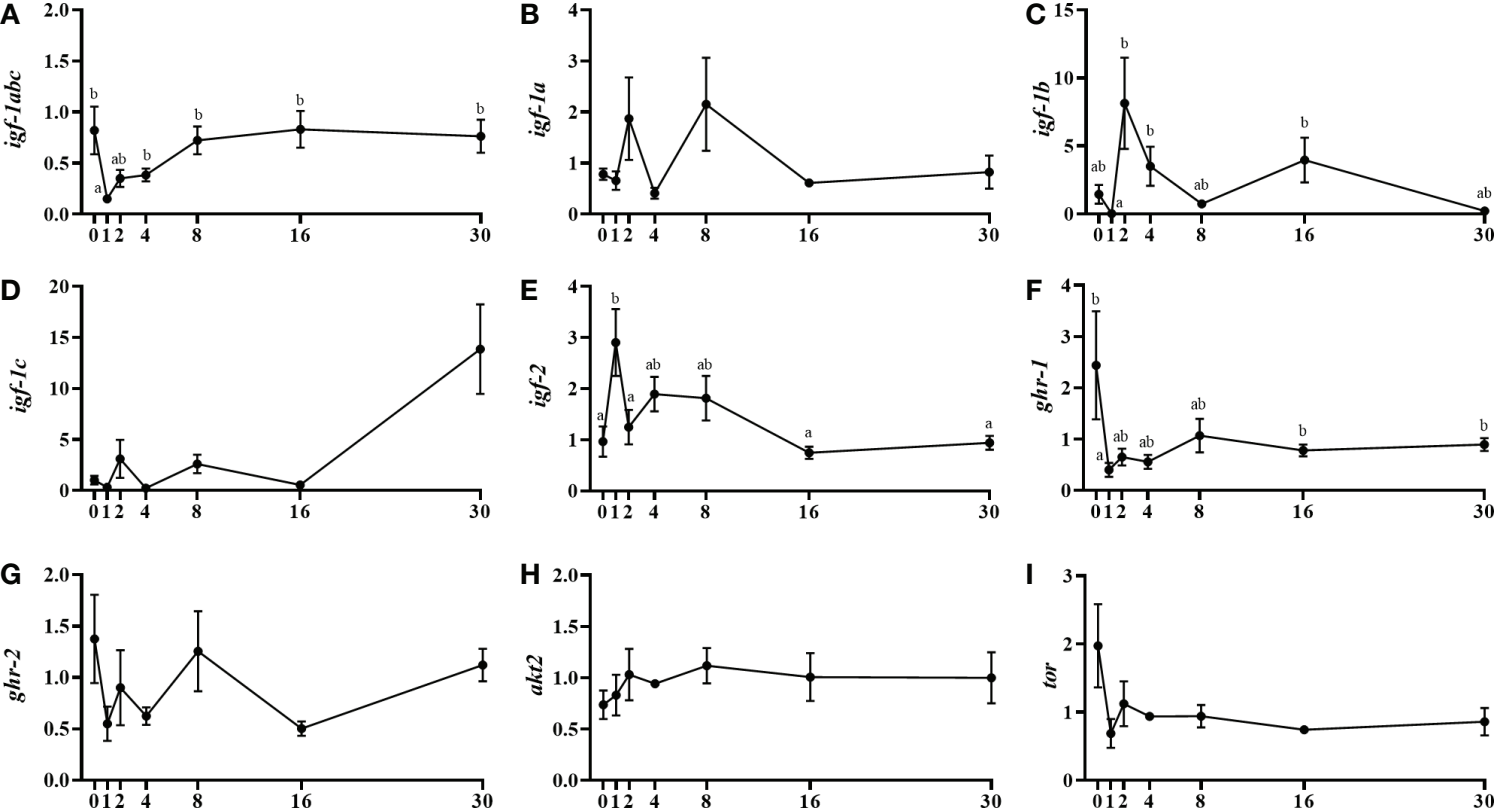
Relative gene expression of the insulin-like growth factors, *igf-1abc* **(A)**, *igf-1a* **(B)**, *igf-1b* **(C)**, *igf-1c* **(D)**, *igf-2* **(E)**, *growth hormone receptors, ghr-1* **(F)**, *ghr-2* **(G)**, and *signaling molecules, akt2* **(H)**, and tor **(I)** in injured epaxial muscle of fish. Data are shown as means ± SEM, n=10. Statistical analysis was assessed by one-way ANOVA. Different letters indicate significant differences of factor time (days post-injury) (Tukey’s HSD or Games-Howell post-hoc test, p < 0.05).

Total Igf-1 mRNA levels (*igf-1abc*) significantly decreased from day 0 to day 1 and, subsequently, increased from day 4 post-injury to recover normal levels that were maintained at day 30 (**Figure 2A**). Concerning the Igf-1 splice variants, *igf-1b* mRNA levels showed a significant increase between days 1 and 2 that was recovered at day 8, while the other two variants (*igf-1a* and *igf-1c*) did not show any clear pattern of expression or significant differences (**Figures 2B–D**). Contrarily, the *igf-2* gene was significantly upregulated from day 0 to day 1 and after that, the expression decreased by day 2 and specially at day 16 remaining low until day 30 (**Figure 2E**). Growth hormone receptors (Ghrs) reported a significant fall in the case of *ghr-1* expression at day 1 and a notable subsequent recovery 16 days after injury, although it was not possible to determine any significant expression change in the paralogue *ghr-2* (**Figures 2F, G**). With regards to downstream signaling molecules, *akt2* gene expression remained constant through the experiment and the mRNA levels of the serine/threonine protein kinase *tor* showed a non-significant tendency to decrease between day 0 and day 1 (**Figures 2H, I**).

### 3.3 Proteolytic systems genes expression during muscle regeneration

**Figure 3:**
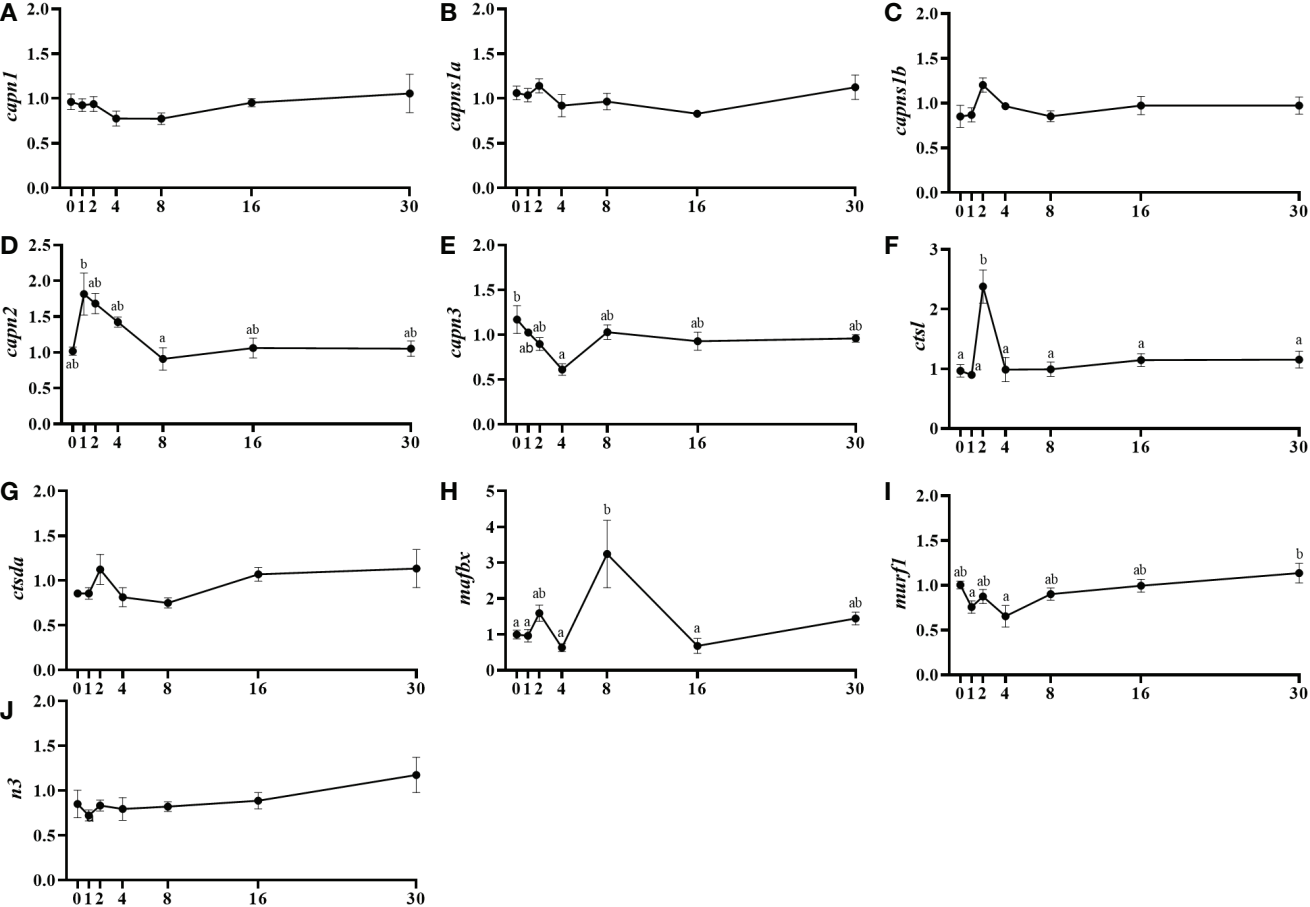
Relative gene expression of the proteolytic systems' markers, *capn1* **(A)**, *capns1a* **(B)**, *capns1b* **(C)**, *capn2* **(D)**, *capn3* **(E)**, *ctsl* **(F)**, *ctsda* **(G)**, *mafbx* **(H)**, *murf1* **(I)**, and *n3* **(J)** in injured epaxial muscle of fish. Data are shown as means ± SEM, n=10. Statistical analysis was assessed by one-way ANOVA. Different letters indicate significant differences of factor time (days post-injury) (Tukey’s HSD or Games-Howell post-hoc test, p < 0.05).

Calpain-1 (*capn1*) and its regulatory subunits (*capns1a* and *capns1b*) showed basal mRNA levels without changes throughout the different days of study (**Figures 3A–C**). On the contrary, *capn2* showed a significant increase in mRNA levels at day 1 then decreasing until day 8 (**Figure 3D**). Then, *capn3* was downregulated from day 0 to day 4 after injury then recovering the initial expression levels by day 8 (**Figure 3E**). Cathepsin L (*ctsl*) presented a significant transient increase of mRNA levels at day 2, but no changes in *cstda* gene expression were observed (**Figures 3F, G**). Regarding the ubiquitin-proteasome system members, *mafbx* significantly increased from day 4 to day 8 to later decrease by day 16, whereas *murf1* showed significantly higher mRNA levels at day 30 compared to days 1 and 4, and the mRNA levels of *n3* were not changed in this study (**Figures 3H–J**).

### 3.4 Gene expression of other local markers during muscle regeneration

**Figure 4:**
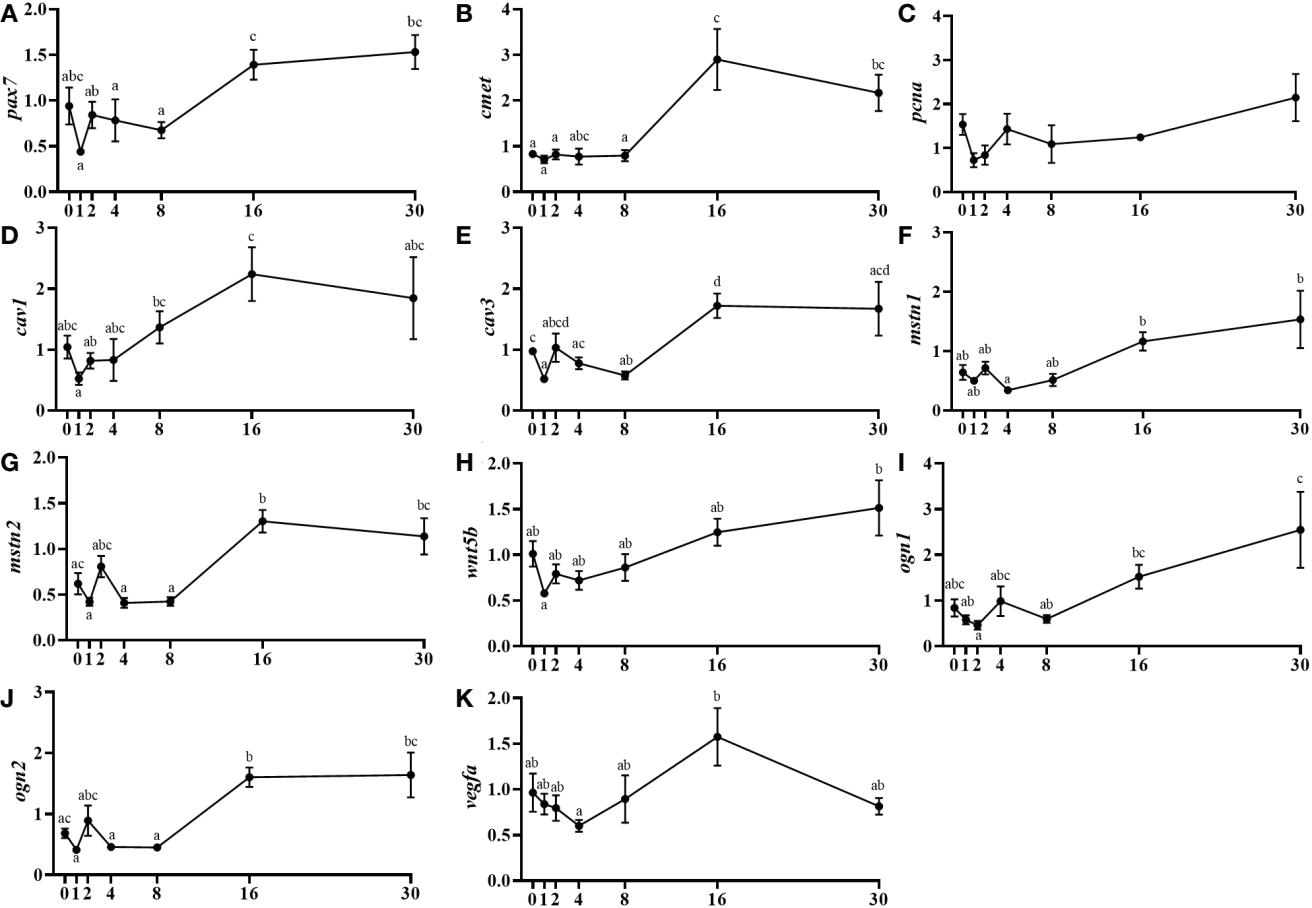
Relative gene expression of other muscle-related markers involved in muscle regeneration, *pax7* **(A)**, *cmet* **(B)**, *pcna* **(C)**, *cav1* **(D)**, cav3 **(E)**, mstn1 **(F)**, mstn2 **(G)**, *wnt5b* **(H)**, *ogn1* **(I)**, *ogn2* **(J)**, and *vegfa* **(K)** in injured epaxial muscle of fish. Data are shown as means ± SEM, n=10. Statistical analysis was assessed by one-way ANOVA. Different letters indicate significant differences of factor time (days post-injury) (Tukey’s HSD or Games-Howell post-hoc test, p < 0.05).

Pax7 and the hepatocyte growth factor receptor (*cmet*) mRNA levels were low until day 8 to then increase significantly at day 16 maintaining the expression elevated until day 30 (**Figures 4A, B**). The *pcna* expression did not present significant changes (**Figure 4C**). The caveolin gene, *cav1* was maintained in a basal expression showing only a significant upregulation at day 16 compared to days 1 and 2 (**Figure 4D**). Moreover, *cav3* showed a similar profile but in this case, the gene expression strongly increased from day 8 to 16 and continued high until 30 days after injury (**Figure 4E**). Concerning the negative regulators of muscle growth, *mstn1* mRNA levels increased progressively from day 4 until day 30, with significant differences at day 16 post-injury (**Figure 4F**). Similarly, *mstn2* mRNA levels showed an upregulation from day 8 to 16 and continued high until the end of the study (**Figure 4G**). The secreted factor *wnt5b*, from the Wnt family, increased gradually its mRNA levels from day 4 until day 30, being significant that rise at 16 days onwards (**Figure 4H**). In the case of the small leucine-rich proteoglycan osteoglycin, muscle mRNA levels of *ogn1* increased significantly from day 8 to 30 and similarly *ogn2* increased from day 8 to day 16 and then remained high until day 30 (**Figures 4I, J**). Finally, for the vasoendothelial growth factor (*vegfa*), minor fluctuations in its mRNA levels were observed, with only a significant increase in expression when comparing between days 4 and 16 (**Figure 4K**).

### 3.5 Inflammatory markers gene expression during muscle regeneration

**Figure 5:**
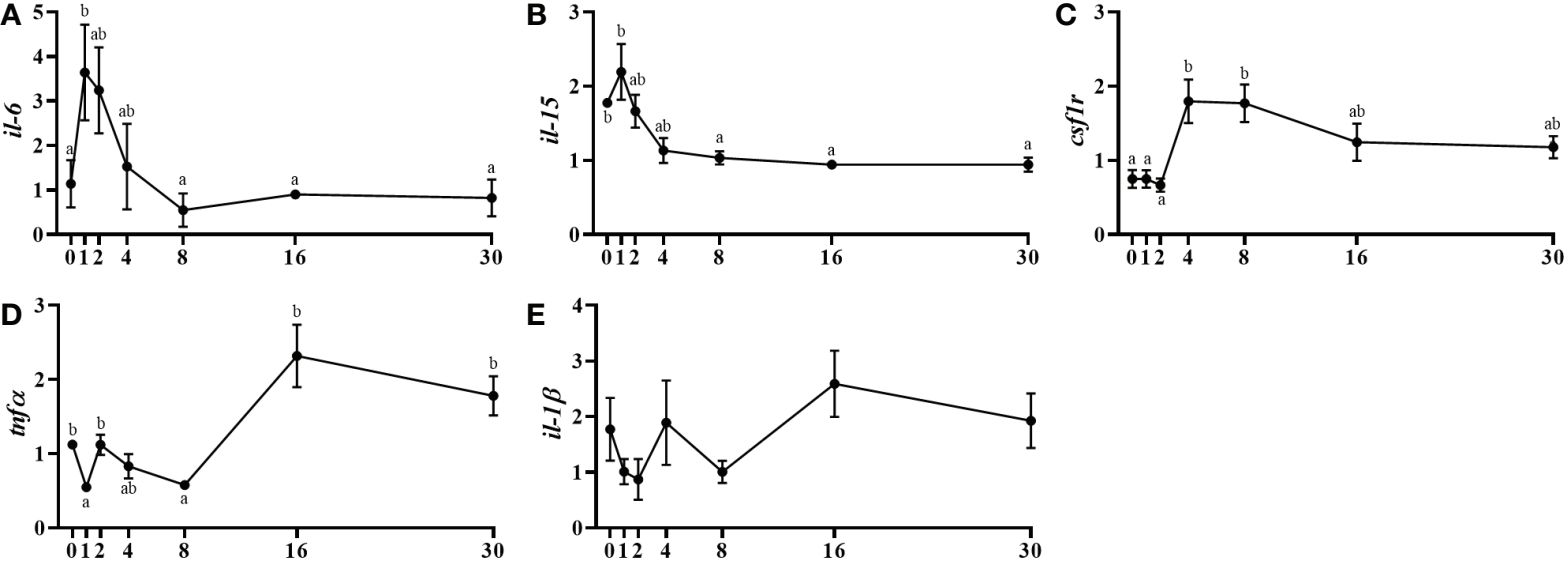
Relative gene expression of the inflammatory markers, *il-6* **(A)**, *il-15* **(B)**, *csf1r* **(C)**, *tnfα* **(D)**, il-1β **(E)** in injured epaxial muscle of fish. Data are shown as means ± SEM, n=10. Statistical analysis was assessed by one-way ANOVA. Different letters indicate significant differences of factor time (days post-injury) (Tukey’s HSD or Games-Howell post-hoc test, p < 0.05).

The gene expression of the pro-inflammatory cytokine interleukin 6 (*il-6*) was incremented significantly at day 1 post-injury and then decreased progressively up to day 8 when the expression returned to significantly lower basal levels (**Figure 5A**). The *il-15* expression was similarly slightly increased at day 1 being those levels significantly higher comparing with days 16 and 30 (**Figure 5B**). The colony-stimulating factor 1 receptor (*csf1r*) gene expression raised significantly from day 2 to 4 and remained high until day 30 (**Figure 5C**). Moreover, *tnfα* showed a significant upregulation at day 16 and continued high until the end of the study (**Figure 5D**) and, the *il-1β* expression did not show any significant change (**Figure 5E**).

### 3.6 Bone markers gene expression during muscle regeneration

**Figure 6:**
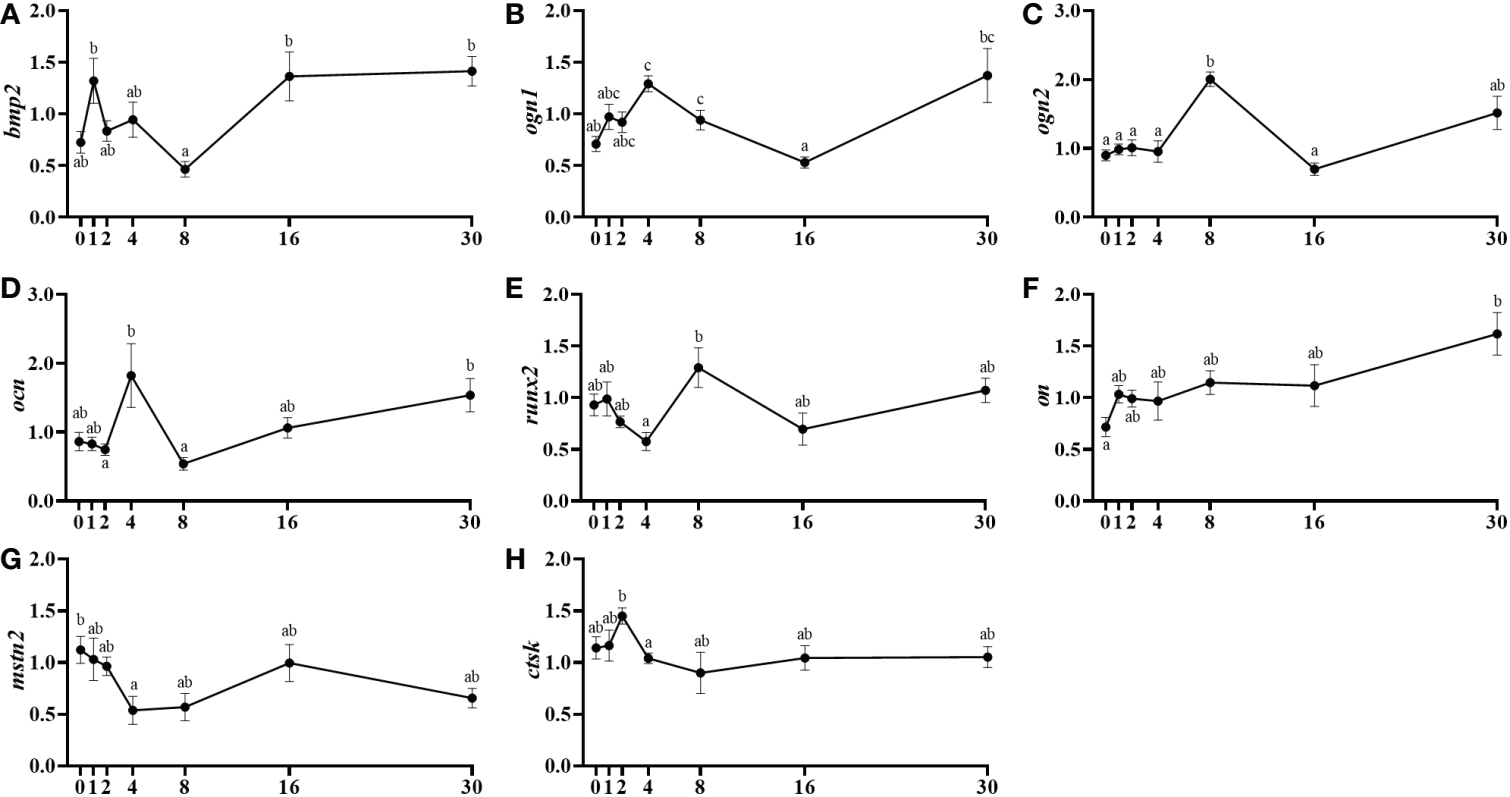
Relative gene expression of the bone markers, *bmp2* **(A)**, *ogn1* **(B)**, *ogn2* **(C)**, *ocn* **(D)**, runx2 **(E)**, *on* **(F)**, *mstn2* **(G)**, and *ctsk* **(H)** in the vertebra of injured of fish. Data are shown as means **±** SEM, n=10. Statistical analysis was assessed by one-way ANOVA. Different letters indicate significant differences of factor time (days post-injury).

The bone morphogenetic protein 2 (*bmp2*) presented a significant mRNA levels reduction from day 1 to day 8 post-injury, followed by an important recovery by day 16 (**Figure 6A**). Concerning the bone mRNA levels of osteoglycin, *ogn1* increased significantly from day 0 to day 4, then its levels decreased in a major way until day 16 and finally, it raised importantly again at day 30 (**Figure 6B**). The *ogn2*, showed a parallel but delayed pattern of expression showing its significantly highest mRNA levels at day 8 (**Figure 6C**). The mRNA levels of the key mineralization marker osteocalcin (*ocn*) slightly increased from day 2 to 4 and then fell significantly at day 8 to finally being recovered at day 30 (**Figure 6D**). The analysis of the transcription factor *runx2* expression showed a significant increment in the mRNA levels between day 4 and day 8, to then slightly decrease again although not significantly (**Figure 6E**). Osteonectin (*on*) mRNA levels increased progressively in a long-term way from day 0 to day 30 presenting significant differences only between those two, initial and final days (**Figure 6F**). Regarding *myostatins* in bone, *mstn1* showed no expression and *mstn2* mRNA levels decreased significantly from day 0 to 4 and then slightly recovered its expression at day 16 (**Figure 6G**). Finally, the mRNA levels of the proteolytic marker cathepsin k (*ctsk*) decreased significantly from day 2 to day 8 after the muscle injury being maintained afterwards (**Figure 6H**).

## 4 Discussion

Juvenile gilthead sea breams were injured in the epaxial white muscle with a 14G needle. Then, muscle histological analysis plus assessment of changes in the transcription of key genes from muscle and bone tissues during a 30-day regeneration period was performed. The histological evaluation revealed at day 1 fibers in the process of necrosis, while 16 days after the injury no traces of damaged or necrotic fibers were observed in the gilthead sea bream. Our results agree with those of Rowlerson et al. (44), who found in the same species that muscle regeneration was weakly detected seven days post-injury, still with some necrotizing fibers, but at 21 days post-lesion many areas were regenerated, and the presence of new young fibers could be observed. In fact, in our study, at day 30 the myotomes showed a complete normal morphology with many new and small muscle fibers present.

The Gh-Igfs axis is the main regulator of muscle growth and contributes to the process of regeneration after tissue injury in vertebrates (20, 45) including fish (46). In this sense, the contribution of Igf-1 during tissue repair has been recognized (47) but the role of Igf-2 is not so well known although its contribution to myogenesis was suggested by demonstrating binding to the Igf-1 receptor, activating the Akt pathway and then MyoD (48, 49). Furthermore, Ge et al. (50) found in mice that *igf-2* mRNA levels also increased drastically during early muscle regeneration. In this study, the peak of *igf-2* at the beginning of muscle repair (i.e., day 1) is consistent with this early and pre-Igf-1 function at the onset of myogenesis. In fact, it has been previously observed in gilthead sea bream myocytes that Igf-2 contributes to muscle regulation mainly in the early stages of development and before Igf-1 (9, 51). Indeed, Igf-1 action takes place slightly later towards the initial stages of myoblast differentiation (52). In agreement with that, in the present study, total *igf-1* expression decreased at day 1, recovering the expression levels at days 4, 8, 16 and 30 after injury, probably mainly due to the upregulation of the splice variant *igf-1b* at day 2 post-injury. Concerning receptors, *ghr-1* expression followed a similar profile to that of *igf-1* with a rapid decrease the first days after injury and a progressive and slow increase afterwards, returning to initial levels at day 16 when the animals recovered from the injury, a response that agrees with its anabolic role in this species (8, 53).

The rebuilding of skeletal muscle after an injury requires a fine regulation of proteinases expression. In that sense, calpains, cathepsins and the UbP are the major systems for muscle protein degradation, and they may contribute to the remodeling of skeletal muscle during regeneration (54). Previously, we have found important transcriptional responses of these proteolytic genes in gilthead sea bream subjected to sustained exercise, different diets, or the combination of both factors (15, 55, 56) or in response to 21 days of fasting (57). In this study, we found at least one gene expressed from each one of the three proteolytic systems at different times during regeneration. In fact, the expression of *capn2* and *ctsl* was increased at day 1 and 2 respectively, while *mafbx* increased at day 8 post-injury and *murf1* at day 30 compared to the early days. In a study done in rats that were injected with 0.75% bupivacaine, a drug that at this concentration produces a chemical injury, the enzymatic activity of Capn1 and Capn2 was maximum at day 5 post-injury, that of Cathepsin L and D at day 3, and the proteasome peaked at day 5 to progressively return to control levels by day 21 (54). This profile of proteolytic gene expression can be comparable to the expression profiles obtained in this study in gilthead sea bream and suggests that the sequence of activation of proteolytic genes during muscle regeneration appears to be quite well conserved through vertebrates. Specifically, calpains seemed to have a more relevant role in proliferation of gilthead sea bream cultured myocytes and the UbP system in cells differentiation (58). In addition, it has been demonstrated that *mafbx* interacts with *myod* regulating its expression; therefore, in this study *mafbx* might be inhibiting *myod* expression prematurely at day 8 in order to ensure the correct activity of Mrfs from day 16 onwards (59). In summary, some of these proteolytic genes could be necessary in our study species, to modulate myogenesis, as observed previously in mammals (60). Thus, these genes could be involved in myoblast proliferation and differentiation (61, 62) but are also required for myoblast fusion (63, 64).

In line with this, as a part of the characterization of the recently identified fusogens Myomaker and Myomixer in gilthead sea bream, aiming to estimate their functions, gene expression of these molecules in addition to the Mrfs were studied under different myogenesis conditions including this model of muscle regeneration (65). All these molecules showed low expression values until day 8, and then started to increase to reach a peak at day 16 post-wound to subsequently slowly decrease to recover initial levels by day 30. Recently, Manneken et al. (66) reviewed the process of muscle regeneration in zebrafish (*Danio rerio*), focusing on the differences to amniotes myogenesis during both, development, and repair. It is noticeable the similarity of the transcriptional profile of the different Mrfs analyzed in that study with those in gilthead sea bream. Moreover, Rowlerson et al. (44) showed that at 7 days post-lesion a weak reaction to desmin antibody appeared only in bigger fibers but not in presumed newly formed fibers; and at 21 days the regeneration was well advanced in many muscle areas. Forcina et al. (67) and Musarò (14) also reviewed the mechanisms regulating muscle regeneration in mammals, indicating that the MRFs participate in the process between 4 and 7 days post-injury, reaching the maturation and functional recovery of the muscle between days 10 and 15. The profile is again very similar to that observed in our previous study in gilthead sea bream (65) although the faster myogenic regeneration process in mammals could be explained by their higher body temperature compared to fish. In fact, in rainbow trout (*Oncorhynchus mykiss*), a species that grows at a lower water temperature than gilthead sea bream, the complete muscle regeneration in a similar experiment took longer (i.e., day 30) and *myomaker* and *myomixer* indeed peaked later, between days 16 and 30 after injury (68).

Other regulatory factors of muscle growth like *pax7*, *cmet*, *caveolins*, *wnt5b, ogn, vegfa* or *myostatins* followed the same tendency, with non-significant small fluctuations up to day 8 but significant increases in expression at 16 and/or 30 days post-lesion. Indeed, the parallelism between the expression profiles of these genes and the Mrfs makes sense due to its recognized role during mammalian myogenesis. For instance, very little is known about the role of caveolins in fish, but the increase observed in *cav1* and *cav3* suggests their participation in fish myogenesis activating SCs as described in mice and humans (26, 69); following *pax7* and *cmet*, factors that are known to stimulate cell cycle activation and proliferation of SCs (21, 22). Moreover, the progressive increase in the expression of *wnt5b* was indicative of its potential role at the end of muscle regeneration during myoblast differentiation and fusion although a larger contribution from this or other members of the Wnt family could be expected in the early stages (28). However, again, the information in fish is very scarce and probably their role is not exactly the same as in mammals (29). The expression of *ogn1* and *ogn2* in the muscle increased from day 8. In a previous study, Costa et al. (70) determined the expression of *ogn* in primary cultures of gilthead sea bream myocytes and observed a significant increase in their expression at day 8 of culture development, at the stage of differentiation and fusion of myocytes to form myotubes. In the present study, it is possible to consider that *ogn* transcripts in muscle are also participating in the fusion of the newly formed fibers. Regarding the expression of *vegfa*, the peak observed at 16 days post-injury is consistent with the main period of new muscle fibers formation plus maturation, and therefore, increased angiogenesis and myogenic factors highest expression; supported by the role previously demonstrated for this factor in muscle remodeling in gilthead sea bream (56). Finally, in the case of *mstn1* and *2*, their gene expression was increased by day 30, which fits with their known role in gilthead sea bream as negative regulators of myogenesis, therefore helping to the completion of the process in this regeneration scenario (71). Thus, the period between days 8 and 16 after the injury seems to be in this species the main period of activation of myogenic factors to induce muscle regeneration. This is a little later that the timing described in mammals but faster than that observed in rainbow trout (67, 68, 72, 73).

In the healing process of a tissue, acute inflammation and immune cells play a critical role in almost all phases of regeneration. Damaged muscle fibers undergoing necrosis induce the infiltration of many types of immune cells (74, 75). Therefore, the infiltrating cells help clearing dead cells from the injured area as well as secrete different types of cytokines to recruit more immune cells in addition to producing cellular responses to regulate muscle cell activation, proliferation, and differentiation (19). In our study, it has been possible to observe a first wave of *il-6* and *il-15* increased expression in the early days after injury. In a mouse model of cardiotoxin-induced muscle injury, Zhang et al. (76) showed how high levels of *il-6* were detected from the first day after injury (24 h) and demonstrated that this cytokine is crucial for macrophage infiltration, and that the lack of it in a knockout mouse model inhibited myoblast proliferation. Moreover, some studies support the fact that Il-6 also induces proteolysis of muscle cells by activating and modulating proteolytic systems (77, 78). On the other hand, *il-15* is highly expressed in mammalian muscle, and it is believed that, in addition to its inflammatory function in a pro-inflammatory context, it may be a myokine as it promotes myogenesis in response to exercise as well as facilitates muscle regeneration (79, 80). The results of our study could be showing such mentioned functions, and in the regeneration in gilthead sea bream both Il-6 and Il-15 could be acting in a pro-inflammatory phase in addition to favoring myogenesis, although we cannot distinguish if these cytokines are specifically expressed in muscle cells, immune cells, or both. Furthermore, Tnfα has been reported to play an important role in muscle regeneration in mammals (81, 82). This cytokine can attract muscle SCs to the damaged site to promote their proliferation and differentiation (83). The increased expression of *tnfα* in gilthead sea bream muscle 16 days after injury could be indicative of this function since its upregulation occurred at the same time as the entire myogenic program was initiated. In fact, the releasing of a whole continuum of pro-inflammatory cytokines into the injured muscle stimulates the infiltration of monocytes that go on to exhibit an inflammatory profile to carry out phagocytosis and then, switch to anti-inflammatory macrophages to stimulate myogenesis (84–86). The colony stimulating factor 1 (Csf1) controls the production, differentiation, and function of macrophages and its receptor (Csf1R) mediates the biological effects (87). In our study, the strong increase detected in the expression of *csf1r* from day 4 on could indicate the arrival to the muscle of macrophages after the wave of pro-inflammatory interleukins. Moreover, the expression of this receptor remains elevated up to day 30, which could mean the maintenance of a population of anti-inflammatory and myogenesis-supporting macrophages until the end of the regenerative process.

Referring to genes expressed in bone, the small leucine-rich proteoglycans *ogn1* and *ogn2* have been characterized in gilthead sea bream during the *in vitro* development of different cell types including bone and muscle (70). Hence, their significantly increased expression in the bone tissue close to the muscle wound at days 4 and 8 post-injury, respectively, in the current study, agrees with their reported upregulation at the onset of osteoblasts as well as myocytes differentiation. Moreover, Tanaka et al. (88) also demonstrated the regulatory crosstalk role between bone and muscle of *ogn*, but references in fish are very poor. In this study, the earlier expression of *ogn* in bone and later in muscle could indicate a stimulus from bone to enhance the recovery of the injured muscle. Similarly, *ocn* expression modulation during regeneration with increased levels at day 4 *versus* day 8, support its role as a promising osteokine participating in the regulation of the muscle response after an injury in fish, as already demonstrated in mammals (89). Indeed, Ocn has been proven to be able to increase importantly muscle activity in elderly mice individuals (90) and to directly promote protein synthesis in myotubes, explaining why this hormone is responsible for muscle maintenance during aging (91). Thus, the increase of *ocn* at day 4 post-injury before the upregulation of Mrfs would support its stimulatory role in fish muscle too. The *bmp2* and *ctsk* genes showed an early activation of expression at days 1 and 2 post-injury, respectively, although *bmp2* expression decreased at day 8 recovering basal levels at the end of the experiment. This early upregulation of *bmp2* could be related to the highest expression found for this molecule at the beginning of embryonic development in gilthead sea bream (92), but also to situations in which bone (and muscle) growth is stimulated, such as for example in Atlantic salmon (*Salmo salar*) reared at elevated water temperatures (93). Moreover, the peak of expression of *ctsk* at day 2 resembled the increase in expression observed after 20 days of fasting (57) suggesting that *ctsk* and therefore, bone resorption, may have a role in muscle remodeling after stressful conditions. The key osteogenic factor *runx2* after a fall at day 4, peaked at day 8, keeping similar levels until the end of the experiment, perhaps suggesting an induction of osteogenesis at this time to support the subsequent muscle mass development. Furthermore, the inter-tissue inhibitory role of Myostatin has been already well characterized in mammals (89, 94). In our model species, a previous study of fasting and refeeding proved how myostatin could act coordinating musculoskeletal growth and suggested its potential role as osteokine in fish (57). In this regeneration study, a regulatory role for myostatin could also be hypothesized, since bone decreased *mstn2* expression by day 4 in response to the muscle injury possibly to allow proper muscle remodeling, thus giving more insights of the importance of myostatin between the bone and muscle crosstalk in fish. Finally, the ECM marker *on* showed a progressive increase during regeneration with the maximum expression reached at day 30 coinciding with the highest expression observed for *on* at the end of *in vitro* and *in vivo* osteogenesis in gilthead sea bream according to its key role regulating the later stages of tissue development (33, 92). In summary, these results open a promising new line of research to study the possible crosstalk between bone and muscle in fish to regulate skeletal muscle development and growth.

## 5 Conclusions

**Figure 7:**
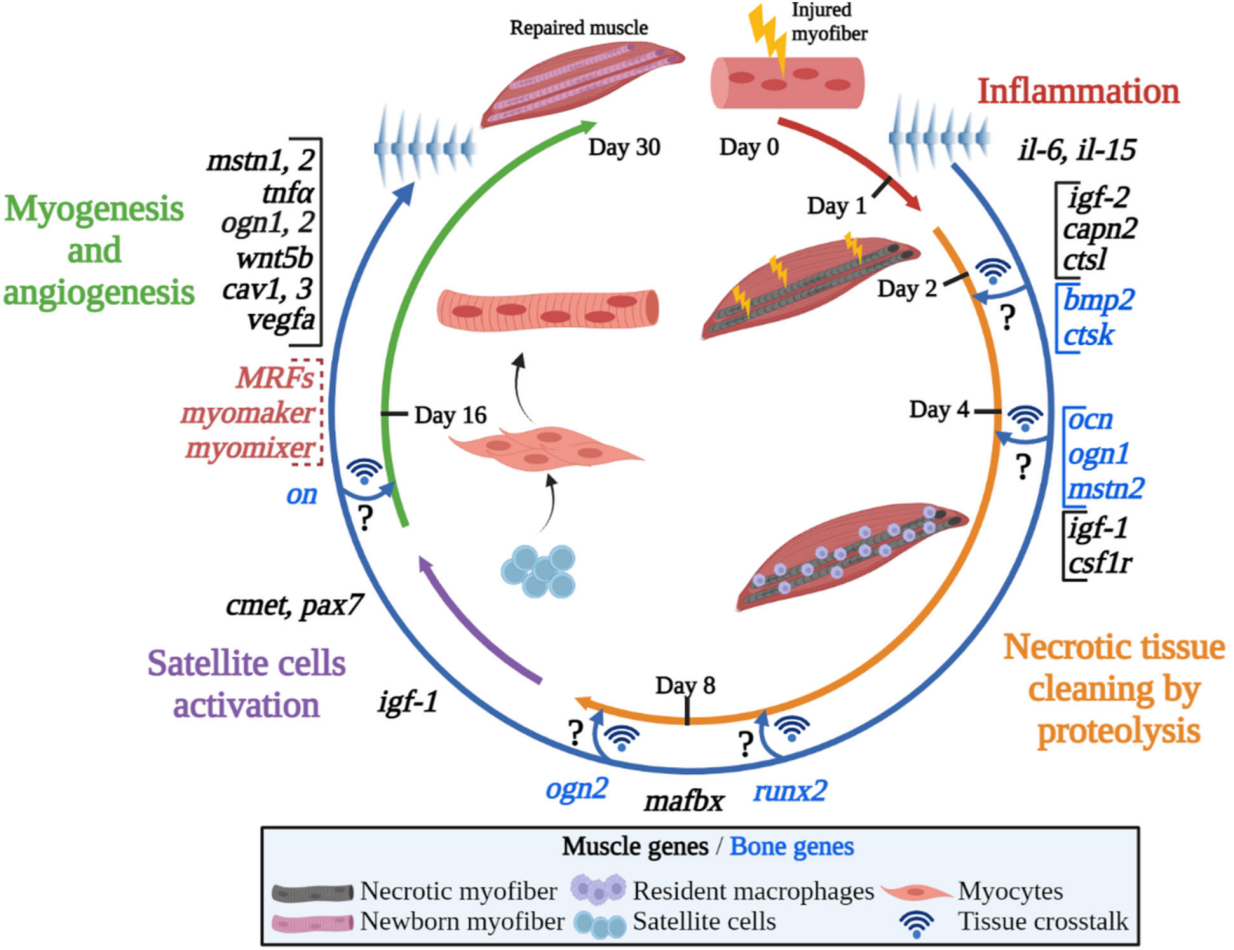
Graphical summary of the proposed model for fish muscle regeneration after an injury. The figure shows an overview of the different processes occurring after an injury in the gilthead sea bream muscle in a circular way, presenting the progressive appearance of the main molecules transcriptionally upregulated/modulated in both, muscle and bone. The circle is divided into the four main steps that take place during the regeneration process, from the initial inflammation and proteolysis to the satellite cells activation and myogenesis. Note that Mrfs, *myomaker* and *myomixer* data has been extracted from (65). The hypothetical muscle and bone interactions suggested are indicated by crossing arrows and question mark symbols. Created with BioRender.com.

In gilthead sea bream, the generation of a wound and the presence of damaged and dead muscle fibers firstly activated pro-inflammatory genes as *il-6* and *il-15* that contributed cleaning the injured tissue and stimulating the muscle regeneration process (**Figure 7**). Then, proteolytic factors, first *capn2* and *ctsl*, and later on the UbP system member *mafbx* allowed an effective degradation of the damaged tissue.

At the very early stage of 1-day post-injury, it was noticeable the increase of *igf-2* and the decreases of *ghr1* and total *igf-1* expression. The return to basal levels at day 8 of *igf-2* was parallel to the increase of *igf-1* and simultaneous to the induction of many other genes’ expression involved in the regulation of myogenesis like *pax7*, *cmet*, *cav1*, *cav3*, and *wnt5b*, indicating the beginning of muscle remodeling. In fact, *vegfa* also showed a clear peak of expression at 16 days post-lesion, which parallels the Mrfs and the fusogens *myomaker* and *myomixer* patterns of expression previously described (65), thus suggesting that progression of myogenesis and angiogenesis is taking place resulting in new fibers formation.

Furthermore, it could be hypothesized that some bone-derived genes such as *bmp2*, *ogn1*, *ocn* and *mstn2* could be participating by sending information to the muscle mainly at the early stages of the regenerative process to control harmonic musculoskeletal growth, thus potentially presenting a role as osteokines in fish.

Overall, this study shows how different mechanisms are orchestrated to recover an injured muscle and allows knowing for the first time in gilthead sea bream the timely contribution of different molecules along the regeneration process of damaged skeletal muscle fibers.

## Data availability statement

The original contributions presented in the study are included in the article/**Supplementary Materials**. Further inquiries can be directed to the corresponding author.

## Ethics statement

The study was conducted according to the guidelines of the European Union Council directive (EU 2010/63) and approved by the Ethics Committee of the University of Barcelona (protocol code CEEA 37/20).

## Author contributions

JG conceptualized the study. AO-T, MP-A, VJ-P, IG-P, AS-M and JG performed the sampling. AO-T, MP-A, VJ-P, FM and IG-P performed the laboratory analyses. AO-T, MP-A, VJ-P and JG analyzed and interpreted the data. IN, EC, JB and JG acquired funding. AO-T, MP-A, IN, EC, JF-B, DG, JB and JG drafted and critically reviewed the manuscript. All authors read and approved the final paper. The authors have declared no conflict of interests. All authors contributed to the article and approved the submitted version.
