# Supplementary material for: Muscle regeneration in gilthead sea bream: Implications of endocrine and local regulatory factors and the crosstalk with bone
Source: Front Endocrinol (Lausanne). 2023 Jan 23;14:1101356. doi: 10.3389/fendo.2023.1101356 (PMC9899866; doi:10.3389/fendo.2023.1101356)
Supplement: Supplementary file 1 [file Table_1.docx]

**Muscle regeneration in gilthead sea bream: implications of endocrine and local regulatory factors and the crosstalk with bone**

**Aitor Otero-Tarrazón**^†^**, Miquel Perelló-Amorós**^†^**, Violeta Jorge-Pedraza, Fatemeh Moshayedi, Albert Sánchez-Moya, Isabel García-Pérez, Jaume Fernández-Borràs, Daniel García de la serrana, Isabel Navarro, Josefina Blasco, Encarnación Capilla and Joaquim Gutiérrez^*^.**

Department of Cell Biology, Physiology and Immunology, Faculty of Biology, University of Barcelona, Barcelona, Spain.

† These authors contributed equally to this work

*** Correspondence:** Joaquim Gutiérrez, jgutierrez@ub.edu; Tel.: +34-934-021-532

**Keywords: skeletal muscle, injury, regeneration, GH-IGFs axis, proteolytic systems, myogenesis, crosstalk, bone.**

**Supplementary material**

**Table S1**

Primers used in the qPCR analyses. F: forward; R: reverse; Ta: annealing temperature.

| Type | Gene | Primer Sequences (5’-3’) | Ta (ºC) | Accession Number |
| --- | --- | --- | --- | --- |
| Reference | *rpl27a* | **F:** AAGAGGAACACAACTCACTGCCCCAC  **R:** GCTTGCCTTTGCCCAGAACTTTGTAG | 68 | AY188520 |
|  | *rps18* | **F:** GGGTGTTGGCAGACGTTAC  **R:** CTTCTGCCTGTTGAGGAACCA | 60 | AM490061.1 |
|  | *ef1α* | **F:** CTTCAACGCTCAGGTCATCAT  **R:** GCACAGCGAAACGACCAAGGGGA | 60 | AF184170 |
| GH-IGFs axis | *igf-Ia* | **F:** AGGACAGCACAGCAGCCAGACAAGAC  **R:** TTCGGACCATTGTTAGCCTCCTCTCTG | 60 | AY996779 |
|  | *igf-Iab* | **F:** AGTCATTCATCCTTCAAGGAAGTGCATCC  **R:** TTCGGACCATTGTTAGCCTCCTCTCTG | 60 | EF688015 |
|  | *igf-Iabc* | **F:** ACAGAATGTAGGGACGGAGCGAATGGAC  **R:** TTCGGACCATTGTTAGCCTCCTCTCTG | 60 | EF688016 |
|  | *igf-II* | **F:** TGGGATCGTAGAGGAGTGTTGT  **R:** CTGTAGAGAGGTGGCCGACA | 60 | AY996778 |
|  | *ghr1* | **F:** ACCTGTCAGCCACCACATGA  **R:** TCGTGCAGATCTGGGTCGTA | 60 | AF438176 |
|  | *ghr2* | **F:** GAGTGAACCCGGCCTGACAG  **R:** GCGGTGGTATCTGATTCATGGT | 60 | AY573601 |
| Inflammation | *il1β* | **F:** GCGACCTACCTGCCACCTACACC  **R:** TCGTCCACCGCCTCCAGATGC | 60 | AJ277166 |
|  | *il6* | **F:** GCTCTGCTGGGTGTGCTCC  **R:** GTCTCCCACTCCTCACCTTG | 60 | AM749958 |
|  | *il15* | **F:** GAGACCAGCGAGCGAAAGGCATCC  **R:** GCCAGAACAGGTTCAAGGTTGACAGGAA | 60 | JX976625.1 |
|  | *tnfα* | **F:** CAGGCGTCGTTCAGAGTCTC  **R:** CTGTGGCTGAGAGGTGTGAG | 60 | AJ413189 |
|  | *csf1r* | **F:** ACGTCTGGTCCTATGGCATC  **R:** AGTCTGGTTGGGACATCTGG | 60 | AM050293 |
| Signaling | *akt* | **F:** GCTCACCCCACTCTTCAGAC  **R:** AAATTGGGAAATGTGCTTGC | 60 | AY996779 |
|  | *tor* | **F:** CAGACTGACGAGGATGCTGA  **R:** AGTTGAGCAGCGGGTCATAG | 60 | EF688015 |
|  |  |  |  |  |
|  |  |  |  |  |
| Muscle growth-related | *pax7* | **F:** ATGAACACTGTCGGCAACG  **R:** AGGCTGTCCACACTCTTGATG | 64 | JN034418 |
|  | *pcna* | **F:** TGTTTGAGGCACGTCTGGTT  **R:** TGGCTAGGTTTCTGTCGC | 60 | AY550963.1 |
|  | *cmet* | **F:** TCCTTCGACATCACCGTGTTC  **R:** ATCATCACTGTGTAGAGGGCGTC | 60 | XM_030426770.1 |
|  | *mstn1* | **F:** GTACGACGTGCTGGGAGACG  **R:** CGTACGATTCGATTCGCTTG | 60 | AF258448.1 |
|  | *mstn2* | **F:** ACCTGGTGAACAAAGCCAAC  **R:** TGCGGTTGAAGTAGAGCATG | 60 | AY046314 |
|  | *vegfa* | **F:** ACACAAGAAGACGGCGAAAG  **R:** ATGAGCCGTTGTTGTTTGCG | 60 | XM_030405264.1 |
|  | *cav1* | **F:** GACGACGTTGTCAAGGTAGACT  **R:** GTACTTGGTGACGGTGAAGGT | 53 | XM_030427009.1 |
|  | *cav3* | **F:** CTGAGGGTGTGGACAAGGTCG  **R:** GTCAGGCCACGATAAACCCA | 55 | LOC115583739 |
|  | *wnt5b* | **F:** TGGTGAGATCTGATTGCAGCGGT  **R:** TCAGAGCGAGCGACCACCAT | 60 | XM_030440162.1 |
| Proteolytic systems | *capn1* | **F:** CCTACGAGATGAGGATGGCT  **R:** AGTTGTCAAAGTCGGCGGT | 56 | KF444899 |
|  | *capn2* | **F:** ACCCACGCTCAGACGGCAAA  **R:** CGTTCCCGCTGTCATCCATCA | 61 | KF444900 |
|  | *capn3* | **F:** AGAGGGTTTCAGCCTTGAGA  **R:** CGCTTTGATCTTTCTCCACA | 56 | ERP000874 |
|  | *capns1a* | **F:** CGCAGATACAGCGATGAAAA  **R:** GTTTTGAAGGAACGGCACAT | 56 | KF444901 |
|  | *capns1b* | **F:** ATGGACAGCGACAGCACA  **R:** AGAGGTATTTGAACTCGTGGAAG | 56 | ERP000874 |
|  | *ctsda* | **F:** CCTCCATTCACTGCTCCTTC  **R:** ACCGGATGGAAAACTCTGTG | 56 | AF036319 |
|  | *ctsl* | **F:** ACTCCTTGGGCAAACACA  **R:** CCTTGAACTTCCTCTCCGT | 54 | DQ875329 |
|  | *ub* | **F:** ACTGGCAAGACCATTACCTT  **R:** TGGATGTTGTAGTCGGAAAG | 54 | KJ524459 |
|  | *murf1* | **F:** GTGACGGCGAGGATGTGC  **R:** CTTCGGCTCCTTGGTGTCTT | 60 | FM145056 |
|  | *mafbx* | **F:** GGTCACCTGGAGTGGAAGAA  **R:** GGTGCAACTTTCTGGGTTGT | 60 | ERA047531 |
|  | *n3* | **F:** AGACACACACTGAACCCGA  **R:** TTCCTGAAGCGAACCAGA | 54 | KJ524458 |
| Bone-related | *runx2* | **F:** ACCCGTCCTACCTGAGTCC  **R:** AGAAGAACCTGGCAATCGTC | 60 | JX232063 |
|  | *bmp2* | **F:** GGAGAAGCAGCGTGGATTAAACACGAAT  **R:** GGCCTGCGCCTCAGTCCAAACATATT | 65 | AY500244 |
|  | *ostc* | **F:** TCCGCAGTGGTGAGACAGAAG  **R:** CGGTCCGTAGTAGGCCGTGTAG | 60 | AF048703 |
|  | *ctsk* | **F:** AGCGAGCAGAACCTGGTGGAC  **R:** GCAGAGTTGTAGTTGGGGTCGTAG | 60 | DQ875329 |
|  | *on* | **F:** AGGAGGAGGTCATCGTGGAAGAGCC  **R:** GTGGTGGTTCAGGCAGGGATTCTCA | 68 | AY239014 |
|  | *ogn1* | **F:** GAAGTCTCTCTTATTCACCTGT  **R:** GTTGTTGGCATTGAAGGAT | 60 | XM_030420705.1 |
|  | *ogn2* | **F:** ATGATGCAACTGAGGACTTTAA  **R:** GCTCCATCTTCAATCTCAG | 60 | KM603668.1 |
